# Challenges posed by COVID‐19 to people who inject drugs and lessons from other outbreaks

**DOI:** 10.1002/jia2.25583

**Published:** 2020-07-01

**Authors:** Tetyana I Vasylyeva, Pavlo Smyrnov, Steffanie Strathdee, Samuel R Friedman

**Affiliations:** ^1^ Department of Zoology University of Oxford Oxford United Kingdom; ^2^ Alliance for Public Health Kyiv Ukraine; ^3^ Department of Medicine University of California San Diego San Diego CA USA; ^4^ Department of Population Health New York University New York NY USA

**Keywords:** people who inject drugs, COVID‐19, infectious disease, outbreak, harm reduction, inequality

## Abstract

**Introduction:**

In light of the COVID‐19 pandemic, considerable effort is going into identifying and protecting those at risk. Criminalization, stigmatization and the psychological, physical, behavioural and economic consequences of substance use make people who inject drugs (PWID) extremely vulnerable to many infectious diseases. While relationships between drug use and blood‐borne and sexually transmitted infections are well studied, less attention has been paid to other infectious disease outbreaks among PWID.

**Discussion:**

COVID‐19 is likely to disproportionally affect PWID due to a high prevalence of comorbidities that make the disease more severe, unsanitary and overcrowded living conditions, stigmatization, common incarceration, homelessness and difficulties in adhering to quarantine, social distancing or self‐isolation mandates. The COVID‐19 pandemic also jeopardizes essential for PWID services, such as needle exchange or substitution therapy programmes, which can be affected both in a short‐ and a long‐term perspective. Importantly, there is substantial evidence of other infectious disease outbreaks in PWID that were associated with factors that enable COVID‐19 transmission, such as poor hygiene, overcrowded living conditions and communal ways of using drugs.

**Conclusions:**

The COVID‐19 crisis might increase risks of homelessnes, overdoses and unsafe injecting and sexual practices for PWID. In order to address existing inequalities, consultations with PWID advocacy groups are vital when designing inclusive health response to the COVID‐19 pandemic.

## 1 INTRODUCTION

An estimated 15.6 million people in the world injected drugs as of 2017 [1]. Most infectious disease research in people who inject drugs (PWID) has focused on blood‐borne and sexually transmitted pathogens, including viruses like human immunodeficiency virus (HIV), hepatitis B and C viruses (HBV; HCV) and bacteria that cause skin and soft tissue infections, endocarditis, tetanus and botulism [2, 3]. The extent to which PWID are vulnerable to COVID‐19, a respiratory disease caused by the SARS‐CoV‐2 virus and associated with a high case‐fatality rate, is yet unknown. Early on in the pandemic, the International Network of People who Use Drugs (INPUD) has published recommendations that include health and wellbeing advice and aim to limit the impact of COVID‐19 on the PWID community [4]. Despite this effort, about half of syringe service programmes in the United States declared a significant reduction in the availability and demand for their services [5]. At the same time, the pandemic led to discovering new ways to provide harm reduction services, like delivering or mailing supplies and using telemedicine hotline for buprenorphine prescriptions [5]. Some programmes also screen their clients for COVID‐19 symptoms, which might help to prevent transmission.

Here we describe why PWID may be more vulnerable to COVID‐19 than other groups and review evidence of other infectious disease outbreaks in PWID that were associated with unsanitary and overcrowded living conditions and compare them to the current pandemic. We also describe short‐ and long‐term challenges that might compromise our efforts to mitigate COVID‐19 epidemic in PWID populations around the world.

## 2 DISCUSSION

### 2.1 PWID vulnerability to COVID‐19

#### 2.1.1 Underlying medical conditions

Older adults, people with asthma (or other lung diseases), smokers, people living with HIV or who are otherwise immunocompromised, and people with hypertension or heart conditions, severe obesity, diabetes, chronic kidney or liver disease are more likely to develop severe COVID‐19 [6, 7, 8]. PWID are disproportionally affected with a number of these conditions. Around 18% of PWID population globally is living with HIV, and HIV‐negative PWID might be immunocompromised as a result of using illicit drugs or due to co‐infection with tuberculosis (TB) or viral hepatitis [9, 10]. Smoking is prevalent in PWID as injecting drugs is often combined with snorting or smoking of crack cocaine, methamphetamine, heroin, cannabis or tobacco [11, 12] Furthermore, opioid use has been shown to adversely affect the respiratory system and cause an array of severe pulmonary defects [13, 14].

#### 2.1.2 Unsanitary living conditions and poor hygiene

Personal hygiene, particularly hand washing, is critical for preventing respiratory infections and requires access to clean water [15]. In drug using populations, the absence of clean water is a common problem and places where drugs are used are often unhygienic [16, 17]. Globally, 22% of PWID experienced homelessness or unstable housing in 2017 (50% ‐ in North America) [1]. While in some locations, like Canada or the United Kingdom, the governments implemented strategies to use empty hotel rooms to house homeless people during the pandemic [18, 19], in many other places PWID might have further reduced opportunities to be housed and gain access to shower and clean water; for example shelters may limit the number of clients in an effort to maintain social distancing.

#### 2.1.3 Difficulties in adhering to quarantine and social distancing

Injecting drugs is often a group activity that includes purchasing, “cooking” and using drugs together, which facilitates pathogens spread in PWID communities [20]. Hepatitis A virus (HAV), an acute infection that rarely leads to liver failure or death, but can be fatal in the presence of chronic liver disease, is transmitted through the faecal‐oral route and thus can be transmitted person‐to‐person among PWID who cook and use drugs together [21]. Similarly, such communal behaviours could create opportunities for “household” COVID‐19 outbreaks that involve large numbers of PWID who use drugs in the same premises. Moreover, many HIV prevention programmes use social networks of PWID to distribute information and invite people to HIV and HCV testing. While being highly effective in reaching the most vulnerable groups of PWID [22, 23], these programmes can potentially increase the risk of COVID‐19 spread by facilitating contacts. Overall, PWID are likely less able to adhere to quarantine and social distancing mandates since they need to seek out other PWID, drug dealers, or harm reduction service providers to procure drugs (in order to avoid withdrawal symptoms), syringes and other paraphernalia. PWID who rely on sex work to support themselves need to seek out clients, whereby social distancing is impossible.

#### 2.1.4 Stigmatization

For COVID‐19, timely hospitalization relative to symptoms onset is critical for positive health outcomes and to reduce transmission [24]. A history of stigmatization at healthcare settings and fear of prosecution and referral to law enforcement make it less likely that PWID seek medical attention for COVID‐19 symptoms [25, 26]. Even if self‐referred to the hospital, PWID might not be admitted, especially if there is evidence of recent drug use [27]. Finally, even if admitted to the hospital, PWID are more likely to leave against medical advice if opioid‐substitution treatment (OST) is not provided [28].

#### 2.1.5 Incarceration

Criminalization of substance use and high rates of incarceration place PWID at high risk of coronavirus infection in correctional institutions, as many jails and prisons combine crowding, poor ventilation and limited access to soap and water [29, 30]. Very early in the COVID‐19 pandemic, outbreaks among correctional facilities staff were reported, and outbreaks among incarcerated people have followed [31]. Poor medical facilities in many jails and prisons mean that morbidity and mortality rates will be exacerbated [29].

### 2.2 Outbreaks of other infections

#### 2.2.1 Hepatitis A virus

Initially HAV outbreaks in PWID were hypothesized to arise from sharing needles [32], but evidence suggests that the standard, faecal‐oral, route of transmission is more plausible [33, 34, 35]. This is due to poor hygienic conditions and possible faecal contamination of drugs (maybe associated with transportation of drugs in the gastrointestinal tract). High rates of HAV infection in groups of PWID have occurred even when they did not inject drugs together, but gathered to smoke or snort [34]. Crucially, those PWID who washed their hands less had higher odds of getting HAV [36].

#### 2.2.2 Tuberculosis

Even though HIV co‐infection is the most important predictor of TB in PWID, drug use was known to be an independent risk factor for TB before HIV was discovered [37]. TB outbreaks in PWID were registered in Europe, the United States and Asia [38, 39, 40, 41]; several outbreaks were specifically identified in substance use treatment facilities or crack houses [42, 43, 44]. PWID are vulnerable to TB due to weakened immune systems (attributed to illicit drug use or HIV co‐infection), crowded and poorly ventilated living conditions and injecting facilities, high prevalence of homelessness and incarceration, heavy alcohol and tobacco use and some common practices that involve sharing of cigarettes or marijuana pipes – all the same factors that might facilitate COVID‐19 transmission [45, 46, 47].

#### 2.2.3 Invasive group A streptococcus

Starting from the 1990s, Invasive Group A streptococcus (iGAS) infection outbreaks caused by clonal strains were reported in PWID in the United States and Europe [48, 49, 50, 51, 52, 53, 54]. Evidence from these outbreaks suggested that bacteria were transmitted person‐to‐person, through respiratory/close contacts, spread by respiratory droplets from colonized patients or asymptomatic carriers, and not through drug contamination [51, 52, 54]. Low hygiene standards, close social interactions and crowded conditions facilitate the spread of iGAS in PWID [53]. Colonized drug dealers who come into contact with large numbers of PWID can be important spreaders of the disease [48].

### 2.3 Immediate challenges

#### 2.3.1 Access to harm reduction

Harm reduction programmes provide clean syringes, HIV and HCV testing, naloxone, OST and other services, aiming to minimize risks from using drugs and slowing down the spread of HIV and HCV. Enforcement of quarantine rules and social distancing made some PWID reluctant to use some of the harm reduction services: in the United States, a decline in HIV and HCV testing was observed since the beginning of the COVID‐19 pandemic due to the fact that these services require direct contact between PWID and staff [5]. Some PWID might find harm reduction sites unreachable due to public transportation bans, which create impossible commute barriers for PWID and staff. Also, the low supply of personal protective equipment for harm reduction programmes puts both staff and clients at risk, further reducing service provision due to staff shortages [5, 55].

#### 2.3.2 Provision of drugs

Border closures, implemented as part of the global COVID‐19 response, have likely disrupted some major drug trafficking routes. Iran, a major heroin trafficking point from Afghanistan to Eastern Africa, was one of the first countries in the world affected by a COVID‐19 epidemic. Multiple countries thus closed their borders with Iran, affecting the drug supply, availability and price [56, 57]. The latter, in turn, can lead some substance users to switch from non‐injection to injection drug use, which is more cost‐effective, and to a rise in unsafe injecting behaviours, as was seen in PWID in Pakistan after the war in Afghanistan in 2001 [58]. Furthermore, some PWID might choose to use oral solutions for injections as has been seen in Australia in early 2000s, causing vein blockage and increasing the risk of pulmonary embolization and hypertension – and further compromising PWID’s lungs in the light of the COVID‐19 crisis [59].

Restrictions in international movement have also significantly affected drug and sex tourism. Thus, an oversupply of heroin can be expected in some locations, as was reported in Mexico following the events of 9/11 in 2001 [60]. Furthermore, as many females who inject drugs often engage in sex work to sustain their substance use, reduction in tourist clients might drive them to riskier sexual practices, for example unprotected intercourse, in exchange for drugs or offers for more money.

At the same time, individual movement restrictions complicate local drug distribution, especially in the settings where street‐based drug purchasing is common. If the drug of choice becomes unavailable, some people might decide to use an alternative, which might result in overdoses due to unfamiliarity with the new drug [57]. Activists lobby drug decriminalization during the lockdowns, and in some countries substitution therapies are being supplied with fewer restrictions, but such response is very limited [18]. In the places where take‐home doses of OST have been made available, accompanying naloxone provision needs to be adequate to prevent overdoses. In other place, reduced access to OST programmes might result in new HIV infections and, again, overdoses. In 2014, after the closure of OST programmes in Crimea following the annexation of the peninsula, around 100 deaths from overdoses were reported among ex‐clients of these programmes [61].

#### 2.3.3 Risk of overdose

Under circumstances of quarantine, PWID are more likely to use drugs alone, which increases their risk of fatal overdose [62]. This is particularly dangerous for opioid users infected with COVID‐19, as they are at a higher risk of breathing difficulties that can lead to fatality [55]. Moreover, PWID might interpret COVID‐19 symptoms for opiate withdrawal, which increases chances of an overdose [63]. Adhering to the rules of quarantine, PWID might also try to procure more drugs than usual to limit their need to obtain drugs on a regular basis; this too might result in overdoses [57]. Alternatively, some PWID might decrease the amount of drugs used during the quarantine, which will decrease their tolerance and thus increase their risk of fatal overdoses after lockdowns are lifted when the access to drugs and drug use practices will return to the pre‐quarantine levels [64].

### 2.4 Long‐term challenges

#### 2.4.1 “Big events” and their economic consequences

Major economic, political, social or ecological crises, often referred to as “Big events”, are emergencies that can result in significant instability [65]. Such “Big events” have been known to have negative effects on health and health‐related behaviours and lead to disease outbreaks and increase in substance use. Some examples include the rise of injection drug use and increased incidence of HIV and TB in the 1990s in Russia and Ukraine after the collapse of the USSR and the rise of HIV in Indonesia after the fall of Suharto dictatorship [66]. The health consequences of “Big events” disproportionately affect poor and otherwise disadvantaged populations, including those using drugs, due to their inadequate access to information, healthcare and their reliance on public services [65]. Since the beginning of the COVID‐19 epidemic in December 2019, health inequalities and their implications have become clear. Even in countries where state insurance is available, financial considerations have affected decisions to seek help due to the lack of paid sick leave and concerns about time lost from work [67]. Financial assistance can offset these concerns, but is often temporary and has not been offered in many developing countries, including those with large PWID populations in Eastern Europe or East and South‐East Asia. Social distancing and travel restrictions have already resulted in a loss of many jobs [68]. For lower socio‐economic strata of the population this means reduced ability to pay rent. Thus, the prevalence of homelessness in people with unstable housing conditions, such as PWID, might grow as it did after the financial crisis of 2007 to 2008 [65].

Post‐2008, public health expenditures also declined, resulting in closures of harm reduction programmes in a number of European countries [65]. As the economic consequences of the COVID‐19 crisis are estimated to be devastating [69], we are likely to observe similar situation post‐2020. The progress made up to date in service provision for PWID might be diminished by reduced service availability and coverage. While governments will try to fight recession, the scale up of services for PWID that have been recently gaining momentum, like naloxone provision or buprenorphine injection depots, might be further delayed.

#### 2.4.2 Access to vaccination

Even when and if a vaccine for SARS‐CoV‐2, the virus responsible for COVID‐19, becomes available, it is unlikely that its uptake in the PWID community will be high. HAV and HBV have been vaccine‐preventable for decades now; but the level of vaccination against these infections remains suboptimal in PWID [70]. This is due to difficulties in accessing PWID with health promotion messages [71], their lack of trust in health professionals [72], and, in many countries, lack of health insurance and an inability to afford the cost of vaccinations [73, 74]. Strategies to increase vaccination uptake in PWID include shortening vaccination schedules, providing vaccination services at local jails, mobile outreach clinics, syringe exchanges or drug rehabilitation centres [21, 75].

## 3 CONCLUSIONS

Global challenges like the COVID‐19 pandemic illuminate unaddressed health inequalities. The burden of the pandemic is the hardest on people with lower socio‐economic status, prisoners and others with inadequate access to health services and information, and those with underlying health conditions – all factors characteristic of PWID. Currently no information exists on the prevalence of COVID‐19 in PWID or what proportion of COVID‐19 cases and deaths come from this group. Similarly, more information is needed on the effect of the lockdown on service provision, and how reduction in HIV and HCV testing will affect incidence in this group. As the fight against COVID‐19 continues, further consultations with INPUD and other PWID advocacy groups are needed when designing inclusive health response to the COVID‐19 pandemic.

## COMPETING INTERESTS

Authors declare no competing interests.

## AUTHORS’ CONTRIBUTIONS

TIV initiated this Commentary and wrote the first draft. PS, SS and SRF contributed equally to the final manuscript. All authors have read and approved the final manuscript.
